# High prevalence of Q fever among patients clinically diagnosed with brucellosis in a Kenyan pastoralist community

**DOI:** 10.3389/fepid.2026.1824266

**Published:** 2026-07-27

**Authors:** Esther Lemarkoko, Pauline Gitonga, Stanley Kang’ethe, Aggrey Keya, Luciano Wairungu, Ryan Musasia, Brian Ogoti, Richard Bowen

**Affiliations:** 1 Department of Medical Services and Public Health, Kajiado County Referral Hospital, Kajiado County, Kajiado, Kenya; 2 Department of Medical Laboratory Sciences, Medical School, Mount Kenya University, Thika, Kenya; 3 Centre for Epidemiological Modelling and Analysis (CEMA), The University of Nairobi, Nairobi, Kenya; 4 Department of Biomedical Sciences, Colorado State University, Fort Collins, CO, United States; 5 Department of Medical Microbiology and Immunology, The University of Nairobi, Nairobi, Kenya

**Keywords:** Brucella, brucellosis, *Coxiella*, diagnostics, fluorescence microscopy, Q fever

## Abstract

*Coxiella burnetii* is a zoonotic bacterial agent responsible for Q fever in both humans and animals. Ruminants are the most common livestock species associated with Q fever infections in humans. The disease presentation in humans range from asymptomatic, non-specific symptoms to fatal illness. In Kenya, no healthcare indicators seek to clinically diagnose and report Q fever because there are no readily available diagnostic technologies. We conducted a prospective observational study with paired serological sampling leveraging on the existing equipment in the Kajiado County referral laboratory to demonstrate antibodies to *Coxiella burnetii* in sera of febrile patients presenting with Brucella-like symptoms using the indirect immunofluorescent assay (IFA) and a fluorescent microscope provided for diagnosis of tuberculosis. A total of 100 paired blood samples were obtained from consenting and assenting study subjects. A pilot-tested questionnaire was used to collect patient's socio-demographic information, knowledge of Q fever disease, and community practices that put them at risk of exposure. *Coxiella burnetii* phase I (IgG) and phase II (IgM) antibodies were characterized, while Brucella spp. IgG antibodies were demonstrated using an indirect enzyme-linked immunosorbent assay (iELISA) and febrile Brucella agglutination test (FBAT). The overall seroprevalence of *C. burnetii* IgG and IgM antibodies was 49% and 27%, respectively, compared to only 13% reactivity with Brucella ELISA. Q fever prevalence substantially exceeded that of brucellosis in patients presenting with brucellosis-like symptoms in this pastoral community, suggesting a substantial burden of undiagnosed Q fever.

## 1 Introduction

Emerging and re-emerging neglected zoonotic diseases continue to pose a significant global public health challenge, particularly in low- and middle-income countries (1, 2). In Kenya, patients exhibiting symptoms consistent with brucellosis, including fever, headache, myalgia, arthralgia, and fatigue, are typically tested exclusively for brucellosis (3–5) despite Q fever presenting with clinically indistinguishable symptoms and sharing identical pathways of transmission from infected livestock (6). This diagnostic approach, often structured around a single-pathogen paradigm, can result in undiagnosed Q fever infections (7). Recent epidemiological evidence suggests that there is substantial circulation of *C. burnetii* in East Africa. A national surveillance study in Kenya revealed a 7.9% seroprevalence in cattle (8). In comparison, studies in pastoralist communities have reported human Q fever prevalence ranging from 20%–35% (9–11). Analysis of Kajiado County syndromic surveillance data from 2018 to 2024 from the Kenya Animal Bio-surveillance System revealed that livestock abortions were the most frequently reported syndrome in the county, with nearly half of these cases occurring in Kajiado Central, specifically in Matapato South Ward, where Meto is located. The laboratory reports obtained for the same period from the Kajiado County Director of Veterinary Services indicated that most aborting animals were strongly positive for immunoglobulin G (IgG) *C. burnetii* antibodies (unpublished). Despite veterinary records and past studies (12) indicating a high seroprevalence of animal Q fever disease, the prevalence of Q fever among humans in the Meto area of Kajiado County is poorly studied.

The burden of Q fever remains undetermined, mainly in clinical practice due to diagnostic limitations (9). The diagnostic gap for Q fever in resource-limited settings has been attributed to the requirement for commercial Q fever ELISA kits or indirect fluorescent antibody (IFA) testing, which necessitates ELISA readers, a fluorescent microscope and trained personnel (13, 14). However, tuberculosis control programmes across sub-Saharan Africa have established extensive fluorescent microscopy networks and trained personnel for immunodetection of detection of Mycobacterium tuberculosis in clinical specimens (15). These microscopes are present in most peripheral county health facilities in Kenya (16). The potential for repurposing fluorescence microscopes primarily used for tuberculosis (TB) sputum smear microscopy for Q fever diagnosis has not been systematically exploited. This study determined the prevalence of antibodies to *Coxiella burnetii* among patients presenting with symptoms of high clinical suspicion for brucellosis in a livestock-keeping community in Kenya, utilizing a standard, commercially-available indirect immunofluorescence assay (IFA).

## 2 Methods

### 2.1 Study site

The study was conducted at Meto Health Centre in Matapato South ward, Kajiado Central sub-county, an area predominantly inhabited by nomadic pastoralists who maintain close and frequent contact with livestock and wildlife, thereby increasing their vulnerability to zoonotic diseases such as brucellosis and Q fever (17). The selection of this site was informed by unpublished veterinary surveillance data from Kajiado County. Laboratory reports from the Kajiado County Director of Veterinary Services (Kajiado-CDVS) for 2018–2024 indicated that most aborting animals were strongly positive for Coxiella burnetii phase I IgG antibodies, and syndromic surveillance from the Kenya Animal Bio-surveillance System (KABS), a national electronic animal-disease reporting system, recorded abortion as the most frequently reported livestock syndrome in the county between January 2018 and June 2024 (3,544 of 15,320 reports, 23%), most commonly in goats and sheep. These findings were supported by a seroepidemiological survey conducted in Kajiado County, which reported substantial seroprevalence of C. burnetii in both livestock and humans, with significant associations to pastoral livelihoods and exposure to animal birth products (18). These combined data underscore the relevance of Meto as a high-risk site for investigating Q fever transmission at the human–livestock–environment interface in pastoralist settings.

### 2.2 Sample size

Sample size was calculated using the formula: *n* = Z^2^ × P(1−P)/d^2^ as described (19), where n is the required sample size, Z is the Z-score corresponding to the desired confidence level (1.96 for 95%), P is the estimated prevalence, and d is the margin of error (0.05). To estimate the prevalence of acute and convalescent Q fever infections, a 95% confidence level (Z = 1.96) and a precision of ±5% (d = 0.05) were assumed. The expected prevalence (P) was derived from the average of two previous human Q fever seroprevalence studies that reported prevalence figures of 26% (12) and 24.44% (95% CI: 21.77–27.26%) (20). The mean of these values, 25%, was used as the expected prevalence. Thus, a target number of 147 paired serum samples was calculated, but due to the nature of the pastoralist population under study, we were not able to obtain that number.

### 2.3 Study design

This was a prospective observational study with paired (acute and convalescent) serological sampling that leveraged human samples collected from outpatients presenting at Meto Health Centre. The study targeted consenting and assenting outpatients aged ≥5 years who presented with fever (≥38 °C) and musculoskeletal symptoms (joint, back, or calf muscle pain), and for whom the attending clinical officer had a high index of suspicion for brucellosis and had referred the patients for Febrile Brucella agglutination Test (FBAT) testing. Additional inclusion criteria included self-reported exposure risk, such as assisting in animal births or consumption of raw milk or blood. Exclusion criteria included children under 5 years of age, patients who declined participation, and those without paired serum samples. A total of 100 acute and convalescent paired samples were collected and analyzed for antibodies to *C. burnetii* phase I or II antigens using indirect immunofluorescence assay (IFA). In conjunction with the laboratory testing, a structured, pilot-tested questionnaire was used to collect demographic data, knowledge and practices related to Q fever, potential exposure risks, and health-seeking behavior. We were not able to obtain data on how patients were treated or long-term follow up. Awareness of Q fever was assessed using a standardized closed-ended questionnaire item that asked whether the participant had previously heard of Q fever. The questionnaire was administered by the study lead (E.S) in the local language.

### 2.4 Laboratory methods

For serologic analyses, 3 mL of venous blood was collected aseptically into clot-activator vacutainer tubes at baseline (acute sample) and again after 14 to 21 days (convalescent sample). Blood samples were processed per national phlebotomy and biosafety guidelines. Sera were separated, aliquoted into labelled cryovials, refrigerated (2–4 °C), and transported to the Kajiado County Referral Hospital under cold chain for storage at −20 °C. Sera were analysed for *C. burnetii* phase I or II antibodies by IFA using commercial test kits (Vircell *C. burnetii* I + II IFA IgG/IgM/IgA; Ref. PCOBU I + II, Abacus dx Spain Ref. PCOBU I + II). The acute sample sera were tested for IgM antibodies against phase II antigens, while the convalescent sample sera were tested for IgG antibodies against phase I antigens; in both cases, the sera were diluted 1:64 in PBS for testing. For detection of IgM antibodies to phase II *Coxiella*, serum samples were pretreated with anti-human IgG sorbent, incubated at 37 °C, and slides stained with FITC-conjugated anti-human IgM. Detection of IgG against phase I antigens involved detection using an FITC-conjugated anti-human IgG second antibody. In all cases, visualisation utilized a Zeiss Primo Star fluorescent microscope at 400× magnification. Positive samples exhibited apple-green fluorescence, indicating the presence of antibodies bound to *C. burnetii* phase I or II antigens, while negative results did not show any fluorescence. Positive and negative controls provided with the Vircell kits were included in every assay to ensure quality control. Sera were screened at a single dilution of 1:64 in PBS, and a positive result was defined as characteristic apple-green fluorescence of the organisms read against the kit controls. All slides were read by the study lead (E.S.), and every reactive (positive) slide was independently re-read and confirmed by a second laboratory colleague (A.K.), with discordant reads resolved by joint review; readers were not blinded to participants’ clinical or exposure status, as the assay was applied as a point-of-care clinical diagnostic test under routine service conditions. As operation labels only, we present IgM-positive reactions for IFA testing of acute sera as indicative of “acute” infection and IgG-positive reactions as cases of “chronic” Q fever; this terminology is discussed below under limitations.

Brucellosis screening was performed using an indirect enzyme-linked immunosorbent assay (i-ELISA, Brucella IgA/IgG/IgM ELISA, Immuno-Biologic Laboratories Inc. IBL *-America-Nr:V* 116.15 –Lot no. EM0045) to detect antibodies to *Brucella* species in serum samples. Sera were also tested for antibodies to *B. abortus* and *B. melitensis* using a FBAT kits with reagents for both *B. abortus* and *B. melitensis* (Biomax Maxwin Health, Tamilnadu, India); FBAT testing has been widely used in Kenya despite reports that the sensitivity and specificity of this test is relatively poor (21, 22). Although not the primary focus of the study, these assays for antibodies to *Brucella* species were used to support or deny clinical suspicion of brucellosis, with i-ELISA providing a confirmatory test based on the manufacturer's defined optical density cut-off values.

### 2.5 Statistical analysis

Categorical variables are summarised as frequencies and percentages, and continuous variables as means with standard deviations or medians with interquartile ranges according to distribution, with normality assessed by the Shapiro–Wilk test. Seroprevalence was estimated for each serological marker separately (recent infection, IgM against Phase II antigen; past exposure, IgG against Phase I antigen) as the proportion of positive results, with Wilson score 95% confidence intervals. Candidate exposures were age, sex, occupation, village of residence, and three pre-specified animal contact variables. Assisted animal birth was dichotomized as ≤1 month vs. >1 month based on the distribution of exposure timing in the cohort, identifying the most recent exposures as the primary risk period, consumption of raw blood during ceremonies, and village-level milk boiling practice. Herd size, a socioeconomic index, and species-specific animal exposure were not recorded. Unadjusted associations with each outcome were assessed by logistic regression, with odds ratios and Wald 95% confidence intervals (Fisher's exact test where cell counts were small). In place of data-driven stepwise selection, which can yield unstable models and biased estimates in small datasets, the adjusted analysis used a single *a priori* specified, parsimonious multivariable model containing the three primary animal-contact exposures (assisted animal birth, raw blood consumption, and village-level milk boiling). This model was fitted using Firth penalized logistic regression to reduce small-sample bias and improve estimation under sparse-data conditions, with profile-penalized-likelihood 95% confidence intervals (23, 24). Candidate demographic variables (age, sex, occupation, and village of residence) were examined in univariable analyses but were not included in the adjusted model because of the limited number of seropositive events, thereby maintaining an appropriate events-per-parameter ratio and minimizing overfitting. Exposed and seropositive counts for all categories are presented in Tables 3, 4 so that the basis and precision of each estimate are transparent. Model discrimination was quantified as the area under the receiver operating characteristic curve and corrected for optimism by bootstrap internal validation with 2,000 resamples, the model being refitted in each resample; apparent and optimism-corrected values are reported. Estimates are interpreted as indicating the direction and approximate strength of association rather than precise magnitudes, and external validation in a larger, independent cohort is recommended. All analyses were performed in R (version 4.3.0; R Foundation for Statistical Computing, Vienna, Austria), using the logistf package for Firth penalized regression, pROC for discrimination, and DescTools for Wilson confidence intervals.

## 3 Results

### 3.1 Participant characteristics

Two hundred acute-phase serum samples were collected between July and December 2023; however, only 100 participants returned for convalescent-phase sampling and were therefore included in the final serological analysis. Demographic and clinical characteristics of these participants are presented in Table 1.

**Table 1:** Participant demographic characteristics and clinical presentation.

Characteristic	Category	Value (*N* = 100)
**Age (years)**	Mean ± SD	46.8 ± 18.3
	Min - Max	5–90
	Median ± SD	46 (32.8–59.3)
**Axillary Temperature (°C) at admission**	Mean ± SD	36.9 ± 0.9
	Min - Max	35.6–39.9
	Median ± SD	36.7 (36.4–37.2)
**Gender**	Female	66
	Male	34
**Village**	Meto	58
	OLoirimirimi	22
	Kumpa	14
	OLdoinyo Orok	6
**Occupation**	Livestock keeper	76
	Crop and livestock farmers	23
	Formal employment	1
**Duration of Illness**	1–3 days	8
	1 week	31
	1 month	30
	3 months	7
	6 months	6
	1 year	18
**Hospital Visits**	1 time	19
	2 times	32
	3 times	26
	>4 times	23
**Fever ≥38 °C at initial examination**	Yes	21
	No	79
**Risk Practices**	Assisted animal birth (Yes)	80
	Raw blood consumption (Yes)	52
	Boil milk (Yes)	91
	Had never heard of Q fever	100

Key demography features of the tested patients were that a majority worked as livestock keepers or mixed crop and livestock farmers, and that high-risk exposures were prevalent: 80% of participants had assisted animal births within the preceding month, and 52% reported consumption of raw animal blood during traditional ceremonies. Protective practices were also common, with 91% reporting routine milk boiling. Notably, 100% of participants reported never having heard of Q fever disease, contrasting with widespread awareness of brucellosis symptoms. Clinical presentation was dominated by musculoskeletal and systemic symptoms. The most frequently reported symptoms were backache (76%), headache (72%), joint pains (67%), and fatigue (51%). At the time of initial examination, 21% of participants had a measured body temperature of ≥38.0 °C.

### 3.2 Q fever IFA

**Figure 1:**
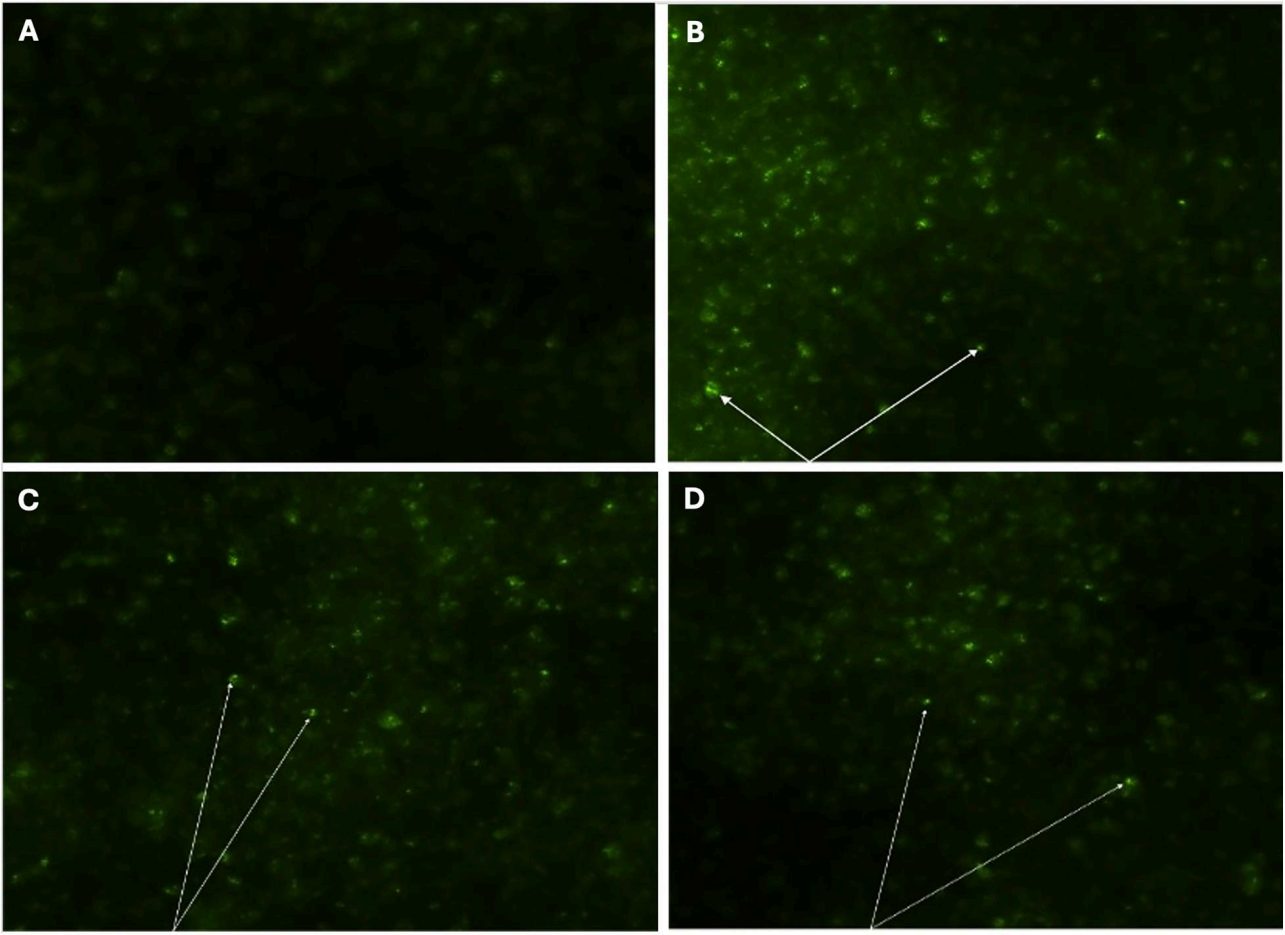
Photomicrographs of Coxiella burnetii spot smears observed using a fluorescence microscope. **(A)** IFA-negative control smear showing absence of specific fluorescence; **(B)** positive control smear; **(C)** IgM-positive patient smear; and **(D)** IgG-positive patient smear. The positive smears show fluorescently labelled C. burnetii organisms displaying the characteristic cocco-bacillary morphology of the bacterium.

Q fever IFA testing was performed using a fluorescent microscopy obtained originally as part of the national tuberculosis diagnostic program. Representative images showing positive and negative Q fever IFA results are presented in Figure 1, illustrating the distinctive fluorescent staining patterns for both IgG Phase I and IgM Phase II antibodies**.**

### 3.3 Comparative pathogen prevalence

Serological results revealed substantial differences in the prevalence of antibodies to *Coxiella* vs. *Brucella* (Table 2). Evidence for *C. burnetii* infections was substantially more prevalent than for brucellosis across all diagnostic markers. IgG antibodies against Phase I antigens. indicating past exposure of undetermined timing to *C. burnetii* were detected in 49.0% of participants (95% CI: 39.0–59.0), while IgM antibodies to Phase II antigens, suggesting recent infection, were found in 27.0% (95% CI: 18.6–36.9) of patients. In contrast, evidence for past or current brucellosis was considerably lower, with iELISA IgG detecting 13.0% positivity (95% CI: 7.7–20.7) and combined FBAT for *B. abortus* or *B. melitensis* showing 10.0% prevalence (95% CI: 5.1–17.6). Individual Brucella species showed even lower prevalence, with FBAT *B. abortus* detecting 6.0% (95% CI: 2.7–12.6) and FBAT *B. melitensis* identifying 4.0% (95% CI: 1.6–9.9). Overall, evidence for past exposure to *Coxiella* was 3.8-fold higher than for brucellosis (ELISA), while indication of recent exposure to Coxiella was 2.7-fold higher than combined brucellosis FBAT prevalence.

**Table 2:** Comparative prevalence of Q fever and brucellosis in patients with livestock-associated febrile illness.

Pathogen	Test	Positive/N	Prevalence (%)	95% CI
Q fever	IgG Phase I (convalescent sera)	49/100	49.0	39.0–59.0
Q fever	IgM Phase II (acute sera)	27/100	27.0	18.6–36.9
Brucellosis	ELISA IgG (acute sera)	13/100	13.0	7.7–20.7
Brucellosis	FBAT *B. abortus* (convalescent sera)	6/100	6.0	2.7–12.6
Brucellosis	FBAT *B. melitensis* (convalescent sera)	4/100	4.0	1.6–9.9
Brucellosis	FBAT *B. abortus or B. melitensis* (convalescent sera)	10/100	10.0	5.1–17.6

**Table 3:** Risk factors for *Coxiella burnetii* IgG seropositivity (past exposure).

Variable	Category	n	Seropositive n(%)	Crude OR (95% CI)	*P*-value	Adjusted OR (95% CI)	*P*-value
Gender	Female	66	31 (47%)	1.00 (ref)	—	—	—
	Male	34	18 (53%)	1.27 (0.55–2.91)	0.573	—	—
Age (years)	5–12	4	2 (50%)	not modelled^a^	—	—	—
	13–24	4	2 (50%)				
	25–59	67	30 (45%)				
	60+	25	15 (60%)				
Occupation	Mixed farming	23	8 (35%)	1.00 (ref)	—	—	—
	Livestock farming	76	40 (53%)	1.85 (0.72–4.75)	0.200	—	—
	Formal employment	1	1 (100%)	not estimable^a^	—	—	—
Assisted animal birth^b^	>1 month ago	26	6 (23%)	1.00 (ref)	—	1.00 (ref)	—
	≤1 month ago	74	43 (58%)	4.62 (1.66–12.86)	0.003*	5.28 (1.87–17.30)	0.003*
Raw blood (ceremonies)	No	48	15 (31%)	1.00 (ref)	—	1.00 (ref)	—
	Yes	52	34 (65%)	4.16 (1.80–9.59)	<0.001*	4.44 (1.85–11.38)	0.001*
Milk boiling^c^	No	9	7 (78%)	1.00 (ref)	—	1.00 (ref)	—
	Yes	91	42 (46%)	0.24 (0.05–1.24)	0.082	0.14 (0.03–0.95)	0.043*

a Not modelled owing to small cell counts.

b Assisted animal birth was dichotomized as ≤1 month vs. >1 month based on seroprevalence patterns (see Results).

c Village-level milk-boiling practice; nine participants reported no boiling. — Demographic variables examined univariable only.

*P < 0.05.

**Table 4:** Risk factors for *Coxiella burnetii* IgM seropositivity (recent infection).

Variable	Category	n	Seropositive n(%)	Crude OR (95% CI)	*P*-value	Adjusted OR (95% CI)	*P*-value
Gender	Female	66	19 (29%)	1.00 (ref)	—	—	—
	Male	34	8 (24%)	0.76 (0.29–1.98)	0.574	—	—
Age (years)	5–12	4	1 (25%)	not modelled^a^	—	—	—
	13–24	4	1 (25%)				
	25–59	67	21 (31%)				
	60+	25	4 (16%)				
Occupation	Mixed farming	23	6 (26%)	1.00 (ref)	—	—	—
	Livestock farming	76	20 (26%)	0.87 (0.31–2.40)	0.790	—	—
	Formal employment	1	1 (100%)	not estimable^a^	—	—	—
Assisted animal birth^b^	>1 month ago	26	4 (15%)	1.00 (ref)	—	1.00 (ref)	—
	≤1 month ago	74	23 (31%)	2.48 (0.77–8.02)	0.127	2.16 (0.75–7.43)	0.189
Raw blood (ceremonies)	No	48	9 (19%)	1.00 (ref)	—	1.00 (ref)	—
	Yes	52	18 (35%)	2.29 (0.91–5.77)	0.079	2.11 (0.86–5.39)	0.113
Milk boiling^c^	No	9	3 (33%)	1.00 (ref)	—	1.00 (ref)	—
	Yes	91	24 (26%)	0.72 (0.17–3.09)	0.659	0.68 (0.17–3.21)	0.610

a Not modelled owing to small cell counts.

b Assisted animal birth was dichotomized as ≤1 month vs. >1 month based on seroprevalence patterns (see Results).

c Village-level milk-boiling practice (nine non-boilers). — Demographic variables examined univariable only.

*P < 0.05.

Evidence for co-occurrence of previous Q fever and brucellosis infections was observed in 13% of participants. This co-occurrence exceeded random expectation (*χ*^2^ = 6.84, *P* = 0.009), consistent with overlapping environmental and occupational exposures. Among co-positive participants, Q fever seroprevalence with brucellosis seroprevalence was most common (8 patients), followed by acute Q fever with brucellosis FBAT (3 patients).

### 3.4 Risk factors for Q fever

Among the 74 participants who had assisted an animal birth, seropositivity for IgG (past exposure) was highest in those with the most recent exposures: 61% (17/28) within 1 week, 75% (15/20) at 1 month, 50% (3/6) at 3 months, 42% (11/26) at 2 weeks, 13% (2/15) at 6 months, and 20% (1/5) at 1 year or longer. This pronounced recency-dependent gradient of seropositivity informed the decision to dichotomize assisted birth as ≤1 month vs. >1 month in subsequent analyses. Risk factor analysis. Univariate and multivariate logistic regression results are presented in Tables 3, 4.

Past exposure (IgG against Phase I antigen). Univariate analysis identified significant associations with assisting an animal birth within the preceding month (OR 4.62, 95% CI 1.66–12.86; *P* = 0.003) and consumption of raw blood during ceremonies (OR 4.16, 95% CI 1.80–9.59; *P* < 0.001). Village-level milk boiling showed an apparent protective association, though the estimate was imprecise due to small numbers of non-boilers (OR 0.24, 95% CI 0.05–1.24; *P* = 0.082). In the adjusted Firth penalized logistic model, both animal-contact exposures remained independently associated with past exposure: assisting animal birth (adjusted OR 5.28, 95% CI 1.87–17.30; *P* = 0.003) and raw blood consumption (adjusted OR 4.44, 95% CI 1.85–11.38; *P* = 0.001). Milk boiling remained protective (adjusted OR 0.14; *P* = 0.043), though imprecise given only nine non-boilers.

Recent infection (IgM against Phase II antigen). The same three exposures were associated in the same direction but of smaller magnitude and did not reach statistical significance: assisting animal birth (adjusted OR 2.16, 95% CI 0.75–7.43; *P* = 0.189), raw blood consumption (adjusted OR 2.11, 95% CI 0.86–5.39; *P* = 0.113), and village milk boiling (adjusted OR 0.68, 95% CI 0.17–3.21; *P* = 0.610). The wide confidence intervals reflect small subgroup event counts. Model discrimination. Bootstrap internal validation (2,000 resamples) assessed optimism in model discrimination. For IgG, the apparent AUC was 0.77, with optimism-corrected AUC of 0.75; for IgM, apparent AUC was 0.64 and optimism-corrected AUC was 0.60. The detailed exposed and seropositive counts are presented in Tables 3, 4.

## 4 Discussion

This study identified significant associations between specific animal-contact exposures and *Coxiella burnetii* seropositivity in a pastoralist community. Assisting with animal births and consumption of raw blood during ceremonies were independently associated with both past exposure (IgG) and recent infection (IgM), while village-level milk boiling showed a protective effect against past exposure. These findings underscore the role of direct contact with potentially infectious animal fluids and tissues in Q fever transmission within pastoralist settings. Among febrile patients presenting at this health centre, 62% were seropositive for *C. burnetii* (any marker). While this reflects substantial Q fever circulation in the pastoral population, this estimate is biased upward by the selection of symptomatic patients and cannot be generalized to the broader community. The 50% loss to follow-up in the convalescent phase and the high-suspicion nature of the enrolled cohort further limit inference about population prevalence. Nonetheless, these findings demonstrate that Q fever is a common exposure in pastoral communities with documented livestock disease.

In the enrolled cohort, 49% had evidence of Q fever past exposure compared to 13% with brucellosis seropositivity. Although brucellosis status was determined by Febrile Brucella agglutination test (FBAT), which has limited sensitivity and specificity (25). This study demonstrated a substantial prevalence of Q fever among patients presenting with brucellosis-like symptoms in a pastoral community, with Q fever prevalence exceeding brucellosis by 3.8-fold. The successful implementation of Q fever testing using tuberculosis programme fluorescence microscopes provides proof of concept for leveraging existing diagnostic infrastructure in resource-limited settings. These findings support the need for changes to current diagnostic approaches in pastoral health systems and indicate an urgent need for protocol revision.

The finding that 49% of patients with brucellosis-like symptoms had evidence of Q fever chronic exposure, compared to only 13% for brucellosis, challenges fundamental assumptions about the distribution of zoonotic diseases in pastoral communities in Kenya. This substantial difference suggests that patients presenting with classical brucellosis-like symptoms are more likely to have Q fever than brucellosis. Yet, current clinical diagnostic suspicion primarily focuses on detecting brucellosis. The clinical implications are profound, as traditional diagnostic approaches in this population would correctly identify 13% of patients with brucellosis while missing 49% with Q fever exposure. This represents a 76% failure rate in zoonotic disease detection among symptomatic patients seeking healthcare. The co-occurrence of Q fever and brucellosis in 13% of participants further complicates clinical management. These patients require treatment protocols that address both pathogens, with Q fever necessitating a more extended treatment duration (18–24 months for chronic infection) compared to brucellosis (6–8 weeks) (26–28). The study also highlights that single-pathogen diagnostic approaches are insufficient in pastoral settings, as they fail to detect the full range of co-circulating infections in febrile patients. As demonstrated in Garissa County, Kenya, febrile outpatients often are affected by multiple bacterial zoonoses, such as Leptospirosis, brucellosis, and Q fever, emphasizing the need for integrated, multi-pathogen diagnostic strategies and coordinated surveillance at the primary healthcare level (7).

The successful implementation of Q fever IFA testing using tuberculosis programme microscopes represents a critical methodological advancement with broad implications for zoonotic disease diagnosis in resource-limited settings. This study demonstrates that an extensive tuberculosis control infrastructure can be effectively repurposed for the diagnosis of Q fever and potentially other infectious diseases. Tuberculosis control programmes have established fluorescent microscopy networks across sub-Saharan Africa, with over 2,000 functional microscopes distributed throughout the healthcare systems (29–31). This existing infrastructure enables rapid diagnosis of Q fever using a well accepted IFA technique at county-level hospitals, reducing reliance on distant reference or research laboratories. As a result, diagnostic turnaround times can be shortened, facilitating earlier clinical decision-making, and ultimately improving patient care in pastoral regions. This represents immediate implementation capacity without the need for new infrastructure, specialized training, or additional maintenance systems.

Recent human serosurveys across East Africa have revealed substantial under-recognition of Q fever. In Kenya, seroprevalence estimates range from 13.2% (32) to 24% (20), with molecular detection rates at 2.2% (14). In Tanzania, paired febrile patient seroprevalence analysis showed acute Q fever rates of 4.7% (2007–2008) and 8.8% (2012–2014) (33). In Ethiopia's Afar region, serosurveillance revealed 25.0% IgG prevalence among pastoralists (10). These regional data underscore that Q fever is a co-circulating pathogen of substantial public health importance in East Africa. The potential scale of Q fever under-recognition has profound implications for clinical management, as current surveillance systems may be misdirected, with resource allocation and prevention strategies optimized for brucellosis control providing minimal impact on Q fever transmission (8).

Limitations.

Several limitations warrant consideration. First, serologic diagnosis to determine the stage of Q fever infection (often referred to as “acute” vs. “chronic”) is clearly problematic, single-sample serology cannot establish infection timing with accuracy, as has been discussed (34–36) and it is not readily possibly to determine with accuracy when initial infection occurred. It would have been beneficial to perform end-point titration of sera for IgG antibodies to phase I antigens, but such was not done. The short interval between paired samples limited assessment of seroconversion dynamics and disease progression. Although the calculated target was 147 paired sera, only 100 were analysed, reducing statistical power particularly for multivariable models; several wide confidence intervals reflect small subgroup sizes rather than strong effects, and adjusted estimates should be read as indicating the direction of association rather than precise magnitudes. Second, participants were enrolled on the basis of clinical suspicion of brucellosis and referral for FBAT, so the sample represents a selected high-suspicion febrile group rather than the general febrile population, which may bias prevalence and association estimates and limits generalisability. Of the 200 patients enrolled, only 100 (50%) returned for convalescent sampling and were analysed; because all participants met identical eligibility criteria and non-return in this mobile pastoralist population is driven mainly by seasonal migration and distance rather than health status, loss to follow-up is likely non-differential with respect to *C. burnetii* serostatus, though selection bias cannot be excluded. Third, brucellosis status was determined by FBAT, which has limited sensitivity and specificity, so the brucellosis figures are approximate and the Q fever–versus–brucellosis comparison should be interpreted cautiously. Fourth, the current study was conducted in a single pastoral region, and generalisability to other epidemiological contexts requires verification. Finally, molecular confirmation of infection by detection of pathogen DNA would strengthen diagnostic certainty but was not feasible in this resource-limited setting.

## 5 Conclusions

This study underscores the critical need to revise diagnostic protocols in pastoral health systems to address the under-recognition of Q fever in sub-Saharan Africa. Implementing dual-pathogen testing should be considered of high importance to improving disease detection and effective patient management. We have demonstrated that screening for Q fever by IFA can readily be established in laboratories that use this same technique and the same equipment used routinely for diagnosis of tuberculosis, with minimal requirement for new training. Furthermore, prioritizing longitudinal research on treatment outcomes and developing combined livestock vaccination strategies for *C. burnetii* and *Brucella* spp. are essential next steps. Embracing One Health approaches that unite human and animal health initiatives can further enhance surveillance and control efforts. These findings provide a scalable model for improving infectious disease diagnostics across similar pastoral settings in the region.

## Data Availability

The raw data supporting the conclusions of this article will be made available by the authors, without undue reservation.
